# How the arts heal: a review of the neural mechanisms behind the therapeutic effects of creative arts on mental and physical health

**DOI:** 10.3389/fnbeh.2024.1422361

**Published:** 2024-10-02

**Authors:** Kelly Sarah Barnett, Fabian Vasiu

**Affiliations:** Balance Medical Center, Vancouver, BC, Canada

**Keywords:** creative arts, emotional regulation, mind-body modalities, music, art, dance, medial prefrontal cortex (mPFC), amygdala

## Abstract

**Background:**

The creative arts have long been known for their therapeutic potential. These modalities, which include dance, painting, and music, among others, appear to be effective in enhancing emotional expression and alleviating adverse physiological and psychological effects. Engagement in creative arts can be pursued as a personal hobby, in a classroom setting, or through a formal therapeutic intervention with a qualified therapist. Engagement can be active (i.e., creating) or passive (i.e., viewing, listening). Regardless of the modality and manner of engagement, the mechanisms explaining the therapeutic efficacy of creative arts remain poorly understood.

**Objective:**

This study aims to systematically review research investigating the neurological mechanisms activated during active or passive engagement in creative arts, with a specific emphasis on the roles of the medial prefrontal cortex (mPFC) and the amygdala in emotional regulation (ER) and creative behaviors. The review seeks to provide preliminary evidence for the possible existence of common neural mechanisms underlying both phenomena, which could inform the development of targeted therapeutic interventions leveraging creative arts for ER.

**Methods:**

A systematic review was conducted following the Cochrane Collaboration guideline and PRISMA standards to identify studies examining the neurological mechanisms underlying creative activities.

**Results:**

A total of six out of 85 records meet the inclusion criteria, with all being basic research studies. Preliminary findings suggest that active and passive engagement with creative arts consistently activate neural circuits implicated in adaptive emotional regulation, including the mPFC and amygdala. These activations mirror the neural pathways engaged in effective ER strategies, suggesting the possible existence of shared mechanisms between creative expression and emotional processing.

**Conclusion:**

The evidence underscores the potential of creative arts as a complementary therapeutic strategy alongside conventional care and other evidence-based mind-body modalities. By elucidating the shared neural mechanisms between creative arts engagement and ER, this review contributes to the theoretical and practical understanding of the role of creative arts in mental health. Future research is recommended to further explore these neural correlations and their implications for therapeutic practice.

## 1 Introduction

### 1.1 Statement of purpose

At present time, there is limited understanding of how the arts heal. On the other hand, we have a better understanding of the areas of the brain that participate in emotional regulation (ER). Having a clearer picture of the neurological relationship between creative behavior and ER can be of use for advancing mental health interventions.

To address current theoretical limitations and potentially improve clinical practice, this study conducted a systematic review of research that directly investigated the neurological mechanisms activated during engagement in or exposure to creative arts (art, dance, and music), with a particular emphasis on studies highlighting the roles of medial prefrontal cortex (mPFC) and the amygdala.

The approach taken in this study is grounded in the hypothesis that engagement in or exposure to creative activities and ER share a common neuronal mechanism involving the mPFC and the amygdala. More specifically, we seek to show, within the limits of the available evidence, that the mPFC and the amygdala consistently activate during engagement in or exposure to art. By doing so, we provide a strong argument in favor of our hypothesis—engagement in or exposure to creative activities and ER lead to the activation of a common neuronal mechanism which includes the mPFC and the amygdala.

By identifying common neural mechanisms, we achieve two things (1) we provide support for the development of a theoretical framework explaining how art heals and (2) we provide knowledge that informs the development of more targeted therapeutic interventions that leverage the benefits of creative arts for ER. This could lead to the design of intervention protocols that are specifically aimed at engaging these neural pathways to maximize therapeutic outcomes.

We begin with a literature review to highlight the current limitations in our understanding of how the arts contribute to ER at the neurological level.

### 1.2 Emotions, feelings, and emotional regulation

The American Psychological Association (APA) defines *emotions* as a complex visceral experience, involving experiential, physiological, and behavioral elements (APA, 2018). Under some definitions, feelings represent a phenomenon that is distinct from emotions. More specifically, feelings can be defined as conscious awareness of emotions (Prinz, 2005).

We know from available research that being connected to and in control of our vast emotional spectrum appears to be an important piece of achieving wellbeing and mental and physical health (Consedine, 2008; Alexander et al., 2021; Menefee et al., 2022); however, significantly more studies are necessary for understanding the mechanisms underlying these observations. Being in control is one way of referring to ER—the processes by which individuals influence which emotions they experience, when they experience them, and how they express and respond to these emotions.

Some theories of emotions, such as evolutionary ones, are rooted in the idea that certain emotions have evolved to serve adaptive functions. These classical theories suggest that basic emotions are biologically ingrained and correspond to innate response programs. Jaak Panksepp, for example, proposed seven different neural pathways underlying different emotions: fear, care, lust, panic, seeking, play, and rage (Panksepp, 1986).

The view of specific brain areas as responsible for specific psychological constructs is contradictable by recent literature. For example Rieck et al. (2024), found that the neural representation of emotion spans multiple regions and cannot be summarized by the activation of a single structure; however, the study did find that neuronal signatures can be used as biomarkers for ER. More specifically, the study aimed to identify a neural signature for cognitive reappraisal using machine learning models in a neurotypical population. Participants viewed neutral and negative images with instructions to either look or reappraise and used functional magnetic resonance imaging (fMRI) to measure brain activity. Least absolute shrinkage and selection operator (LASSO) principal component regression and linear discriminant analysis were employed to classify images based on brain patterns. Key brain regions identified included the prefrontal cortex, insula, cerebellum, and occipital lobe.

In the human brain, as well as in other species, the emotional visceral experience originates in the lower primitive brain: the limbic system. For instance, a human and a cat can both experience fear thanks to their amygdala if they hear a loud noise; humans, however, are capable of not letting their fear dominate their behavioral and physical state, i.e., the cat runs away, but the human, although experiencing sensations of fear, may stay in one place, understanding that there is no immediate threat. This difference is due to humans' conscious awareness of emotions and the ability to name and regulate them. This ability appears to be primarily located in the brain's higher advanced cortices, specifically the prefrontal cortex (PFC). These two emotional processing brain centers or neural networks (i.e., the amygdala and the PFC) have been otherwise referred to as the limbic “low road” and the PFC “high road” (Fishbane, 2007).

Under the definition of feelings as conscious awareness of emotions, we can say that ER can occur when we experience feelings. In other words, the distinction between emotions and feelings under this definition is important because it underscores humans' capabilities of engaging in self-regulation, thus altering their physical and behavioral responses to emotions. The more we understand about the mechanisms that participate in ER, the more capable we should be of controlling them.

Does the self-regulated human have a healthier mental and physical response than the cat? Sometimes. ER plays an important role in mental and physical wellbeing, with advances in the understanding of it suggesting that incorporating ER practices can improve existing mental health interventions (Moltrecht et al., 2021); however, processes that make ER possible can also lead to negative outcomes. In other words, not all “high roads” are beneficial to our health, and many can relate to the harmful effects of unprocessed negative emotions. For example, chronic stress has known negative effects on mental and physical health (Mariotti, 2015; Shangkhum and Zikos, 2023). A frequent cause of chronic stress is habitual negative thinking or unhealthy ways of processing emotions, i.e., rumination (Renna et al., 2020).

Alongside standard-of-care approaches to mental health like psychotherapy and medication, mind-body techniques can counteract stress response (Jung et al., 2010; Wolever et al., 2012; Cozzolino et al., 2020; Kim et al., 2013). The body-mind model of art therapy posits that creative expression facilitates the activation, reorganization, growth, and reintegration of the self through integrated body-mind processes (Czamanski-Cohen and Weihs, 2016). The REPAT study, for instance, demonstrated significant improvements in emotion processing among women coping with breast cancer, highlighting the potential of art therapy to modulate emotional responses through creative engagement (Czamanski-Cohen and Weihs, 2023).

Some mind-body modalities (MBM) that have been shown to improve psychological and physical wellbeing are mindfulness, yoga, and meditation (Kwon and Lee, 2022). Although traditional MBM helps regulate the stress response, these techniques do not necessarily get to the root of why internal chronic stress may be there in the first place. Approaches focused on healthily releasing repressed painful emotions are an alternative solution that directly targets the source of stress. For instance, Emotional Awareness and Expression Therapy (EAET) is surfacing as a newer treatment modality, with two randomized control trials (RCT) showing EAET superior to Cognitive Behavioral Therapy (CBT) for chronic pain (Yarns et al., 2020; Lumley et al., 2017).

Other models of therapy applied to artistic engagement include the expressive arts therapy model (Malchiodi, 2003) and narrative therapy (Rice, 2015). The arts therapy model posits that engaging in different forms of creative expression can help individuals explore and process emotions, leading to improved mental health and wellbeing. Narrative therapy is based on the assumption that creating and analyzing personal narratives through writing or other creative means allows reframing experiences and developing more empowering perspectives.

### 1.3 Creative arts and art therapy in ER

#### 1.3.1 The relationship between ER and creativity

ER can be either adaptive, creating a direct benefit, or maladaptive, creating dysfunction in our mind and body (Gordon and Mendes, 2021). ER mechanisms that are maladaptive (i.e., rumination, suppression) and adaptive (i.e., reappraisal) have been correlated with their consequential deleterious and positive health benefits, respectively. Long-term use of maladaptive mechanisms is correlated with poor mental health and somatic symptoms (Gordon and Mendes, 2021; Salazar Kämpf et al., 2023; Ouhmad et al., 2023; Centers for Disease Control Prevention, 2020; Cavicchioli et al., 2021). fMRI studies have shown that different regions of the brain are dedicated to adaptive and maladaptive emotional processing. For instance, within the prefrontal cortex, the frontal (ventro) medial gyri are involved with adaptive emotional processing mechanisms (Murakami et al., 2015; Hermann et al., 2014; Cutuli, 2014; Gross and John, 2003).

Adaptive ER like reappraisal, which is re-interpreting a negative scenario in a more positive light, has been shown to access neural circuitry on a different “high road” and a different amount of “low road” amygdala activation than maladaptive mechanisms like emotional suppression (Gross and John, 2003). Specifically, reappraisal, mindfulness, and expression of negative emotions have been shown to use the mPFC neural “high road” in conjunction with primal emotional brain centers like the amygdala. Emotional suppression, on the other hand, uses the dorsal PFC (dPFC) “high road” with decreased amygdala activity (Murakami et al., 2015; Hermann et al., 2014; Cutuli, 2014; Ahmed et al., 2015; Etkin et al., 2011; Chen et al., 2017). Depression severity has also been linked to blunted amygdala activity (Ferri et al., 2017). Therefore, adaptive emotional processing has been shown to engage the amygdala more than unhealthy mechanisms like chronic emotional suppression. This may mean subjects are not allowing themselves to access suppressed feelings like fear, which over the long term can have health consequences including all-cause mortality (Murakami et al., 2015; Chen et al., 2017; Chapman et al., 2013).

The activation of the ventromedial prefrontal cortex (vmPFC) with limbic regions is important for healthy ER, stress resilience, and moral judgment (Hänsel and von Känel, 2008; Suzuki and Tanaka, 2021; Hu and Jiang, 2014) and using adaptive emotional high roads i.e., problems solving, positively affects the nervous system, cortisol levels (hypothalamic-pituitary-adrenal axis), and mental and physical health compared to the negative effects of maladaptive high roads, i.e., disengagement (Ahmed et al., 2015; Kop et al., 2011; Gilbert et al., 2017; Patel and Patel, 2019). For instance, one study showed improved regulation of negative emotion with higher vmPFC and improved diurnal rhythm of circulating free cortisol (Urry et al., 2006). Another study showed that vmPFC activation correlates with the activation of the parasympathetic nervous system, improves health outcomes, and plays a pivotal role in biopsychosocial processes of disease (Hänsel and von Känel, 2008).

The evidence above suggests that (1) ER plays an important role in wellbeing defined as positive outcomes in physical and mental health and (2) at least in part, art works by enhancing ER. The clinical relevance of the relationship between ER and art warrants a further investigation of the neuronal mechanisms participating in creative behavior under the assumption of common neuronal mechanisms underlying both phenomena.

#### 1.3.2 Arts in everyday activities and therapeutic settings

Creative arts encompass a broad range of activities including painting, music, dance, and writing, pursued primarily for personal expression, enjoyment, and cultural engagement. These activities are often self-directed and can be undertaken by anyone regardless of their skill level, with the primary goal being creative expression and personal satisfaction (Stuckey and Nobel, 2010).

Personal engagement with creative arts refers to activities pursued as hobbies or for educational purposes in settings such as classes or community groups. This type of engagement is typically self-directed, with individuals choosing activities based on personal interests and goals. Personal engagement can occur individually or in groups. We do not have knowledge of any study which has compared the psychological effects of individual vs. group engagement in creative arts in clinical or non-clinical settings.

There are two modes of art engagement: active participation and passive reception. Active participation consists of creating art and passive reception involves experiencing art created by others. To the best of our knowledge, there are no studies which have investigated whether the clinical effects associated with each engagement modality are explained by the same or different mechanisms or whether the effects in questions are comparable qualitatively and/or quantitatively.

Creative arts have been a vehicle for expression and healing across cultures since ancient times. For example, Egypt's oldest dance, the Zar, was performed for the sole purpose of healing (El Guindy and Schmais, 1994). Engaging in activities such as painting, writing, dancing is embraced not only for personal fulfillment but also for their capacity to modulate emotions. More specifically, evidence suggests that these practices influence mood, promote self-compassion, and enhance overall psychological wellbeing, impacting health at immediate, mid-term, and long-term levels (Stuckey and Nobel, 2010; Fancourt et al., 2019; Graham-Pole, 2000; Ebert et al., 2015; Morris et al., 2005; Šimić et al., 2021; Verger et al., 2022; Mastandrea et al., 2019). For instance, daily creative activities have been shown to improve health markers including cortisol diurnal patterns, autonomic balance, and overall quality of life across various demographic ranges (Stuckey and Nobel, 2010; Kaimal et al., 2016; Bolwerk, 1990; Okada et al., 2009; McCrary and Altenmüller, 2021).

In therapy, creative arts are integrated within different psychotherapeutic modalities to address specific psychological or physiological issues. Examples include art therapy, music therapy, and dance-movement therapy. A recent theoretical development in this field is the body-mind model of art therapy (Corsetti, 2021), which focuses on how art therapy facilitates activation, reorganization, growth, and reintegration of the self through body mind processes. The body-mind model encourages empirical research to explore and validate the key mechanisms posited to underlie the therapeutic effects of art therapy.

Creativity appears to be supported by complex, interconnected brain networks rather than isolated brain regions (Beaty et al., 2014). Engaging in creative tasks may stimulate similar integrative neural processes. For example, there appears to be a link between creative ability to the expression of genes associated with synaptic plasticity (Orwig et al., 2021). These genes are involved in synaptic assembly and signaling, underscoring that the ability to generate novel ideas is related to the brain's capacity to form and reorganize synaptic connections. In therapeutic settings, this suggests that creative arts might foster neural adaptability, potentially aiding recovery or development in clinical populations by promoting neural plasticity. Interventions that enhance synaptic plasticity might be particularly beneficial in rehabilitation settings, where recovery of cognitive functions requires neural reorganization. This assumption is supported by one study with adult art students documenting structural changes like the modification of white matter in response to learning and practicing art, which correlates with increased creative output (Regev and Cohen-Yatziv, 2018).

Clinical studies also support the effectiveness of different creative modalities in improving specific clinical outcomes when integrated within psychotherapy. Here are some examples:

- **Art therapy:** Research suggests that art therapy can improve symptoms in clinical populations. For example, one paper reported that engaging in art making and creative writing improved lab values in dialysis and HIV patients (Stuckey and Nobel, 2010). Art has been shown to significantly reduce cortisol levels irrespective of an individual's prior skill in healthy subjects (Kaimal et al., 2016). Systematic reviews suggest art therapy has clinical effects on mental disorders including anxiety, depression, Alzheimer's, and autism (Hu et al., 2021; Schlegel et al., 2015).
- **Music therapy:** Music therapy has been shown to be effective in improving vital signs, myocardial oxygen demand, and reducing anxiety after acute myocardial infarctions (Bolwerk, 1990). Music therapy can enhance parasympathetic tone and reduce catecholamines and cytokine levels in elderly patients with cardiovascular diseases and dementia (Okada et al., 2009). Reviews and meta-analyses indicate that music therapy significantly reduces stress-related biomarkers across various patient populations (McCrary and Altenmüller, 2021; Salamon et al., 2003; De Witte et al., 2022; Toyoshima et al., 2011).
- **Dance therapy:** Dance movement therapy (DMT) appears to be an effective intervention for decreasing depression and anxiety while increasing quality of life, interpersonal skills, and cognitive functions. Effects have been reported to remain stable or even improve at a 22-week follow-up (Koch et al., 2019).

#### 1.3.3 The mPFC and amygdala as central components of a neural circuitry underlying both creativity and ER

Creativity is not merely a singular cognitive function but a complex interplay of multiple cognitive processes such as cognitive flexibility, working memory updating, and inhibitory control. These processes are supported by structural and functional networks within the brain, including the PFC, which plays a crucial role in creative ideation and problem-solving. Dopaminergic (DA), noradrenergic (NE), and serotonergic (5-HT) systems—modulate these cognitive functions. For instance, DA pathways are linked to both the enhancement of creative drives and the modulation of cognitive flexibility, essential for novel idea generation. In contrast, NE and 5-HT systems play roles in regulating mood states and motivational aspects of creativity, influencing how environmental and internal stimuli are processed creatively (Khalil et al., 2019).

Studies show that creative activities modulate and regulate emotions, increase empathy and tolerance, influence our moods, and affect our mental and physical health (Fancourt et al., 2019; Fancourt and Ali, 2019); however, understanding how they regulate emotions is still a work in progress. There may be a surfacing neuroscientific model to explain this.

With creativity being such an integral part of our humanity, it arguably makes sense that our brain has wiring dedicated to the creative process. Is this wiring limited to the theorized partition of the right creative vs. left logical brain? Interestingly, no. Creativity is much more expansive across neural networks, using more gray and white matter than once thought.

Along with limbic structures like the amygdala (Chan et al., 2023), there are three neural networks utilized during the creative process: the imaginative or Default Mode Network (DMN), the Central Executive Network (CEN), which engages the conscious brain to think and maintain attention, and the Salience Network (SN), which switches between the DMN and CEN (Raichle, 2015; Beaty et al., 2018a,b; Bressler and Menon, 2010; Bolwerk et al., 2014).

Research suggests the existence of dysfunctional interactions within and between the brain's core neurocognitive networks. For example, Menon's triple network model proposes that disruptions in the DMN, SN, and CEN are critical in the manifestation of psychopathological symptoms across a broad spectrum of disorders (Menon, 2011). In this model, the SN plays a key role in detecting and responding to salient stimuli. Dysfunction in this network can lead to inappropriate salience attribution, which is evident in conditions like schizophrenia where it may contribute to the emergence of hallucinations or delusions. In anxiety disorders, hyperactivity in the SN may lead to exaggerated threat perception and worry. Alterations in the DMN, which is involved in self-referential thought processes, have been implicated in a variety of conditions. For example, hyperconnectivity within the DMN is observed in depression, correlating with rumination and negative self-focus. Conversely, disruptions in the DMN connectivity are noted in Alzheimer's disease, reflecting difficulties with memory and self-referential cognition. The CEN supports higher cognitive functions such as working memory and executive control. Dysfunctions in this network often manifest in cognitive deficits across many psychopathologies, including attention deficit hyperactivity disorder (ADHD) and schizophrenia, where there may be a breakdown in cognitive control and attention regulation. The positive clinical effects on creative arts may be at least partly explained by the effects on the mentioned neurocognitive networks.

If creativity has been shown clinically to improve ER, could there be a common neuronal mechanism underlying both processes? Emotions and creativity appear to be tightly linked and that the neural mechanisms underlying creativity may depend on emotional states (McPherson et al., 2016). Two prominent regions seen to participate in both creativity and adaptive emotional processing are the mPFC, which is part of the DMN, and the amygdala.

##### 1.3.3.1 mPFC in emotional processing and creativity

Both imagination and creativity have been shown to highly engage the DMN, including the mPFC (Abraham, 2016). For instance, a study on the neural substrates of spontaneous musical performance showed that improvisation in music suppresses the “inner critic” or dorsal PFC while activating the mPFC (Limb and Braun, 2008; López-González and Limb, 2012). The mPFC activity in the DMN may play a role not only in spontaneous thoughts and self-referential mental activity but also foster a sense of personal identity and lay the foundation for long-term goal pursuit (Fox and Christoff, 2018). Another relevant study argues that free-flowing complex music results from internally generated self-expression (via the mPFC) and attenuation of activity in the dlPFC (López-González and Limb, 2012). Yet another study on musical creativity concluded that the DMN and intensity of emotional experience may coordinate the drive to create music (Bashwiner et al., 2016). Additional research shows that the DMN or imagination phase of creativity engages the mPFC, and this engagement is related to openness to experience, increased resilience, and increased emotional awareness (Beaty et al., 2018b; Bolwerk et al., 2014; Wei et al., 2014; van Leeuwen et al., 2022; Vaisvaser, 2021).

fMRI studies have shown that healthy emotional processing utilizes the mPFC (Li et al., 2014). This anatomical correlation may suggest that creativity improves emotional wellbeing by utilizing similar “high road” neural networks involving the mPFC of the DMN. One study supports this hypothesis by showing less mPFC activity when using maladaptive discharge to process negative emotions in males, and more mPFC activity when women used an adaptive diversion mechanism to distract from negative emotions (Carlson et al., 2015). In short, being creative helps to process difficult and negative emotions in a healthy way, potentially by activating adaptive emotional neural networks involving the mPFC. Further research in this area could advance a theoretical model that would inform practices underlying the assumption that creative arts can improve emotional, mental, and physical health by improving ER.

Exposure to artistic creations also engages the mPFC. For instance, neuroimaging evidence indicates that activity in the mPFC is linked to viewing aesthetically pleasing images (Cattaneo et al., 2020; Kreplin and Fairclough, 2013) and listening to music (Carlson et al., 2015). This acknowledgment warrants the need to explore the neuronal correlates of both creative engagement and artistic exposure in order to elucidate the role of the mPFC in emotional processing following active or passive exposure to art. Understanding the role of the mPFC in emotional processing during both active and passive engagement would be a step toward elucidating whether the clinical effects associated with each engagement modality are explained by the same or different mechanisms.

Given that engagement in and exposure to creative activities activates the mPFC, we can speculate that creative arts may enhance emotional wellbeing by utilizing adaptive emotional networks. Understanding this connection could lead to more effective interventions for emotional and cognitive disorders.

##### 1.3.3.2 “High road” and “low road” activations in creativity

Creativity activates the limbic “low road” regions of the brain, including the amygdala, which is the seat of primary emotions like fear and joy (Šimić et al., 2021; Bashwiner et al., 2016). Adaptive emotional processing has been shown to engage the amygdala more than unhealthy mechanisms like chronic emotional suppression (Murakami et al., 2015; Chen et al., 2017). Given the activation role of creativity, one can speculate that engaging in creative behaviors may help to engage the amygdala in emotional processing and improve emotional wellbeing, perhaps more so for those who have blunted activity or engage in chronic suppression.

The roles of the mPFC and the amygdala in regulating emotions through creative arts can be understood as complementary yet distinct, based on their involvement in adaptive and maladaptive emotional processing mechanisms. The mPFC appears to be primarily engaged in adaptive ER strategies such as reappraisal, where it facilitates positive reinterpretation of negative experiences by accessing “high road” neural circuits. In creative arts, the mPFC may play a role in processes where individuals manage emotions and foster resilience by processing emotions through self-expression and reflection.

On the other hand, the amygdala, part of the “lower road,” appears to be more directly involved in the immediate emotional response, particularly emotions like fear and joy. Creativity activates the amygdala, which can help individuals access and process these primal emotions, leading to improved emotional wellbeing. In contrast, maladaptive ER strategies, such as suppression, involve reduced mPFC and amygdala activity, potentially leading to poorer mental health outcomes. Therefore, in the context of creative arts, the mPFC may help in reappraising and cognitively managing emotions, while the amygdala contributes to the visceral, emotional experience of the creative process.

By systematically exploring the neural correlates of creative engagement and emotional processing, we can determine whether the activations of the mPFC and amygdala during ER are indeed the same as those activated during passive or active creative engagement. This assessment could help us better understand the relationship between creativity and ER at the neuronal level and potentially inform the development of targeted interventions that leverage creative arts for enhancing emotional wellbeing.

## 2 Methodology

### 2.1 Protocol

The review process was conducted following the guidelines established by the Cochrane Collaboration, which include steps for identifying and selecting studies, extracting data, evaluating the quality of the data, and analyzing the findings (Deeks et al., 2023). Additionally, the reporting of the review adhered to the PRISMA Guidelines (Page et al., 2021).

### 2.2 Eligibility

1. Inclusion criteria:

- Peer-reviewed studies.
- Research presenting empirical data on the neurological mechanisms that participate in (1) ER and (2) creative arts.

2. Exclusion criteria:

- Studies not involving neuroimaging techniques.
- Studies in languages other than English.
- Studies not focused on mPFC or the amygdala.
- Non-human studies.

Our criteria were selected to directly support our aim of assessing whether the mPFC and the amygdala are activated during active or passive creative arts engagement. As such, we excluded studies not involving the brain structures nor neuroimaging techniques of interest.

### 2.3 Search strategy

The literature search was conducted across multiple electronic databases, namely PubMed, PsycINFO, and Google Scholar. The following search queries were entered using Boolean operators (AND, OR) to combine the keywords:

- **Creative arts:** (“art therapy” OR “dance therapy” OR “music therapy” OR “creative arts” OR ‘creative writing”).
- **ER:** (“ER” OR “affect regulation”).
- **Neuroimaging/neurological pathways:** (“neuroimaging” OR “fMRI” OR “PET scan” OR “EEG” OR “medial prefrontal cortex” OR “MPFC” OR “amygdala” OR “neural pathways”).

The search was conducted by combining these groups of keywords using Boolean operators as follows:

- (“art therapy” OR “dance therapy” OR “music therapy” OR “creative arts” OR “creative writing”) AND (“ER” OR “affect regulation”) AND (“neuroimaging” OR “fMRI” OR “PET scan” OR “EEG” OR “medial prefrontal cortex” OR “MPFC” OR “amygdala” OR “neural pathways”).

Filters were applied to limit the search results to peer-reviewed articles, English language, and human studies.

### 2.4 Data collection and quality assessment

- Initial Screening: Titles and abstracts of studies retrieved using the search strategy were screened by one reviewer to identify studies that potentially meet the inclusion criteria.
- Full-text Review: Full texts of the selected studies were obtained and independently assessed for eligibility by one reviewer.

The methodological quality of the included studies was assessed using the Cochrane Risk of Bias tool for randomized controlled trials (Deeks et al., 2023). The risk of bias for each study was assessed by the reviewer using the Cochrane Collaboration's tool for assessing risk of bias (Deeks et al., 2023). This assessment covered various domains, including selection bias, performance bias, detection bias, attrition bias, and reporting bias. Each domain was judged as “low,” “high,” or “unclear” risk.

## 3 Results

### 3.1 Overview

**Figure 1:**
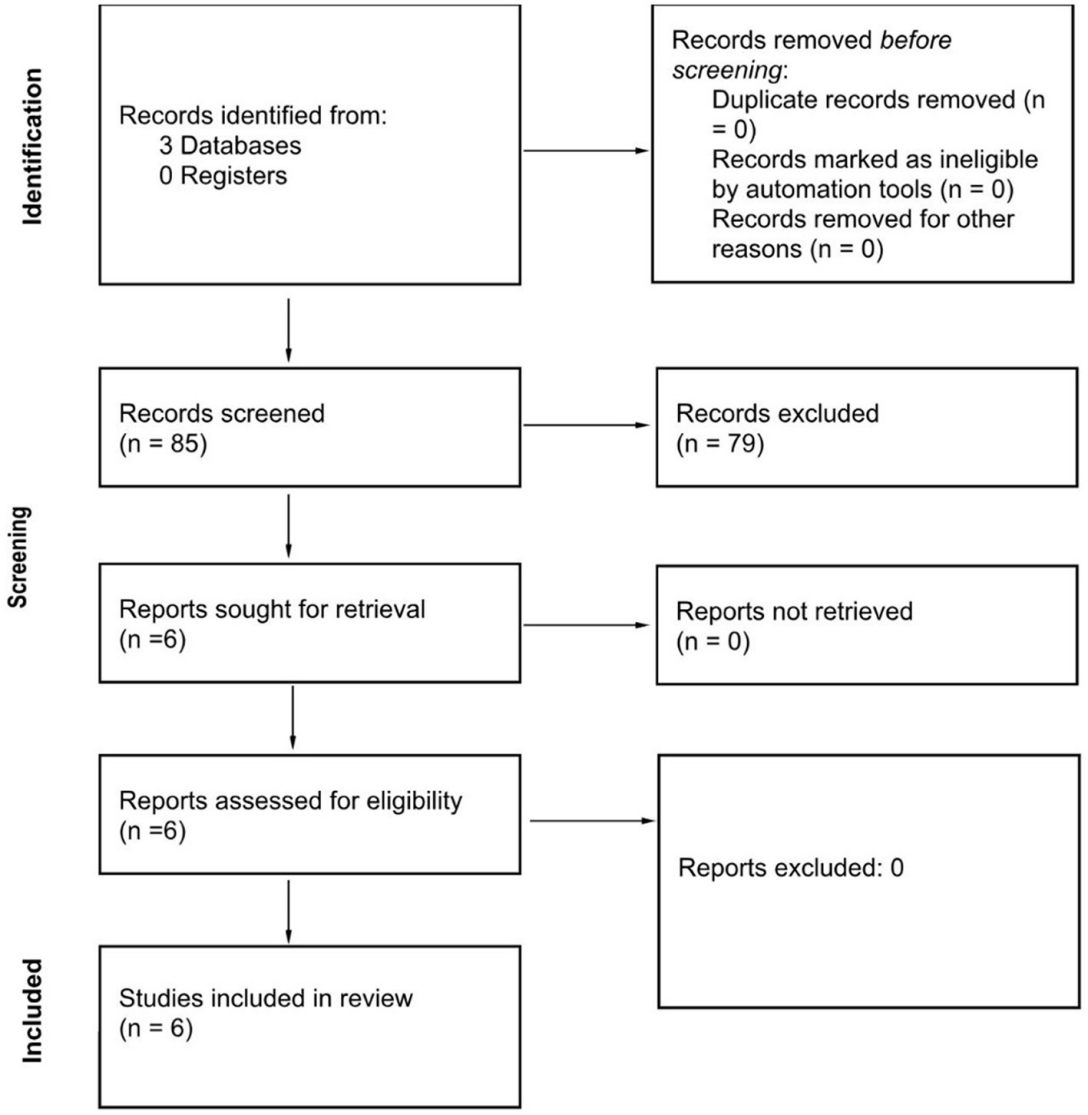
Study selection process.

Three databases were consulted, yielding a total of 85 records. Prior to screening, no records were removed for reasons such as duplication or ineligibility as indicated by automation tools. Consequently, 85 records underwent a screening process. Of these, 79 records were excluded for not meeting the inclusion/exclusion criteria, leaving six reports that were sought for retrieval. All six retrieved reports were then assessed for eligibility, and none were excluded from this stage (Figure 1).

We included studies that investigated the effects of creative arts on ER during both engagement in and exposure to creative arts (Table 1). Three of the studies included were focused on the ER effects of various creative art forms, including drawing, visual art viewing, and music listening. One study did not discuss creative arts but the effects of exposure to music. This study was included on the assumption that exposure to music may at least partly replicate the effects observed during actively listening to music or even music-making. One study examined the neural mechanisms of creativity during creative improvisation but not as part of art therapy. This study was included on the consideration that during therapeutic arts, the client goes on a creative journey following prompts from the therapist. Similarly, in this study, the musician was given the prompt to express himself freely on the piano, which may mean the activation of similar neural mechanisms.

**Table 1:** Summary of studies.

**References**	**Study focus**	**Creative arts discussed**	**ER outcome**
Zhang et al. (2023)	Investigate the ER effects of drawing, comparing venting vs. distraction strategies	Drawing	Emotional states modulated by drawing.
Kreplin and Fairclough (2013)	Examining rPFC activation using fNIRS during viewing of art intended to induce emotions	Viewing visual art	Emotional responses to visual stimuli
Carlson et al. (2015)	Investigates emotion regulation through music and neural responses in the mPFC	Music listening	Discharge strategy
McPherson et al. (2016)	Examines the influence of emotional cues on creativity during piano improvisation	Piano improvisation	Emotionally targeted musical improvisation
Bolwerk et al. (2014)	Investigates effects of visual art production vs. cognitive art evaluation on brain connectivity and resilience	Visual art production and evaluation	Self-awareness and emotional control
Limb and Braun (2008)	Investigated the neural substrates that underlie spontaneous musical performance	Jazz improvisation	Deactivation of limbic structures involved in regulating motivation and emotional tone

The studies employed either functional near-infrared spectroscopy (fNIRS) or fMRI to investigate the neural correlates of ER through art creation or exposure to artistic creation (Table 2). None of the studies included took place in a psychotherapeutic or other clinical setting. Key findings revealed both shared and distinct neural mechanisms involved in processing emotions through creative activities. However, limitations such as sample size, demographic diversity, and the specificity of the observation periods were noted across the studies.

**Table 2:** Neuroimaging technique, key findings, and limitations.

**References**	**Neuroimaging techniques used**	**Key findings**	**Limitations**
Zhang et al. (2023)	fNIRS	Distraction drawing resulted in a higher valence immediately after the activity compared to venting drawing. During relaxation, venting drawing showed greater improvement in valence. Higher PFC was associated with distraction drawing	Limited observation period; lack of consensus on brain activation interpretations
Kreplin and Fairclough (2013)	fNIRS	Increase in rPFC oxygenated blood during positive image viewing	Viewing conditions not manipulated to investigate the gateway hypothesis
Carlson et al. (2015)	fMRI	Discharge strategy related to decreased mPFC activity in males and increased anxiety and Neuroticism. Diversion strategy related to increased mPFC activity in females	No explicit measures of non-musical mood regulation behavior; potential Type I errors
McPherson et al. (2016)	fMRI	Emotional cues modulate neural activity in PFC and limbic areas during improvisation. Positive improvisation associated with greater deactivation of the dlPFC and increased activation in the left amygdala. Negative improvisation linked to increased connectivity between the insula and substantia nigra	Limited sample size; only male participants
Bolwerk et al. (2014)	fMRI	Visual art production enhances functional connectivity between the PCC/preCUN and the frontal cortex (most notably the mPFC), linked to increased self-awareness, emotional control, and psychological resilience	The study only included post-retirement adults
Limb and Braun (2008)	fMRI	Spontaneous musical improvisation leads to a dissociated pattern of activity in the PFC, extensive deactivation of dorsolateral and lateral orbital regions, with focal activation of the mPFC cortex. Changes in PFC activity were accompanied by widespread activation of neocortical sensorimotor areas and deactivation of limbic structures	Study limited to six highly trained jazz musicians

A bias risk assessment across the studies indicated various levels of uncertainty in selection, performance, detection, attrition, and reporting biases (Table 3). Some studies showed a high risk in certain areas, while others maintained low-risk profiles. The overall assessment suggests a need for caution in interpreting the studies' findings due to these potential biases.

**Table 3:** Risk of bias.

**References**	**Selection bias**	**Performance bias**	**Detection bias**	**Attrition bias**	**Reporting bias**
Zhang et al. (2023)	Unclear	High	Unclear	Unclear	Unclear
Kreplin and Fairclough (2013)	Unclear	Low	Low	Unclear	Unclear
Carlson et al. (2015)	Medium	Low	Low	Unclear	Low
McPherson et al. (2016)	Low	Unclear	Low	Unclear	Low
Bolwerk et al. (2014)	Unclear	Low	Low	Unclear	Low
Limb and Braun (2008)	Low	Low	Low	Unclear	Low

### 3.2 Main findings

The study conducted by Zhang et al. (2023) using functional near-infrared spectroscopy (fNIRS) investigated the ER effects of drawing, comparing venting (expressing emotions) and distraction (drawing neutral objects) strategies in 44 college students. After inducing fear through a video, participants were asked to draw based on their emotional experience (venting group) or draw a house (distraction group), followed by a relaxation period.

The study aimed to explore the potential differences in ER between venting and distraction strategies through drawing. Using fNIRS, the researchers measured prefrontal cortex activity, correlating it with self-reported emotional states before and after the drawing activity and a subsequent relaxation period. Initially, the distraction group showed higher emotional valence compared to the venting group immediately after the drawing activity, indicating a quicker recovery from the induced negative emotion. However, no significant difference in emotional valence between the two groups was observed after the relaxation period. During the drawing period, increased activation in BA10 (mPFC) and BA46 (dlPFC) was associated with lower valence (lower emotional improvement). During the relaxation period, decreased activation in these channels was associated with higher valence, suggesting that reduced cognitive control facilitated better emotional recovery.

The study reported a significant difference in mPFC activation between the venting and distraction groups, with the venting group showing a 1.2 μM increase in oxygenated hemoglobin compared to a 0.8 μM increase in the distraction group (*p* < 0.05). The correlation between mPFC activation and emotional valence was *r* = −0.45 (*p* < 0.05), indicating a moderate negative relationship.

The findings suggest that while distraction strategies may offer a quick fix in emotion regulation, venting strategies might lead to a more profound and lasting emotional recovery, evidenced by the changes in emotional valence post-relaxation. The study's findings suggest that expressive art activities engage neural networks involved in adaptive ER where the mPFC and the amygdala play a significant role. The authors noted limitations such as the short duration of the observation period post-drawing activity and the potential for the experimental manipulation (i.e., instructions for drawing tasks) not fully capturing the participants' actual engagement in venting or distraction strategies. Future research is suggested to extend the observation period and explore the underlying mechanisms common to both strategies that facilitate ER.

Bolwerk et al. (2014) investigated the impact of engaging in visual art activities on the brain's functional connectivity, particularly focusing on the DMN, an area which, as discussed earlier, includes the PFC and the amygdala. Utilizing fMRI, the research explores how visual art production (actively creating art) and cognitive art evaluation (cognitively analyzing art) influence psychological resilience and neural connectivity in a non-clinical sample of 28 post-retirement adults. Participants were randomly assigned to either a visual art production group where they actively created art, or a cognitive art evaluation group where they engaged in the cognitive evaluation of artworks at a museum. The DMN's connectivity was analyzed using fMRI before and after the participants attended weekly art sessions for 10 weeks. Psychological resilience was also assessed using the brief German version of the Resilience Scale (RS-11).

The visual art production group showed significant improvement in functional connectivity of the DMN, particularly between the PCC/preCUN and the frontal cortices (BA 8, 9, 10, 46). There was enhanced connectivity from the left and right PCC/preCUN to areas including the premotor cortex (BA 6), prefrontal cortex (BA 8, 9, 10, 46), superior and inferior parietal lobules (SPL, BA 7; IPL, BA 39, 40), and superior and middle temporal gyri (MTG, BA 21; STG, BA 22) at T1 compared to T0.

The cognitive art evaluation group showed weaker improvements in DMN connectivity, with significant connectivity from the right PCC/preCUN to the SPL (BA 7) and PCC (BA 31), and no significant changes for the left PCC/preCUN at T1 compared to T0.

The increases in connectivity were quantified using *z*-scores, with the most substantial improvements observed in the left PCC to left SPL connectivity (*z* = 3.28, *p* < 0.001). The art production group also showed a significant increase in resilience scores (measured by the RS-11 scale), with an average increase of 2.15 points (*p* < 0.01).

This increased connectivity in the art production group was associated with psychological resilience, suggesting that creating visual art may bolster psychological resilience in adulthood. The cognitive art evaluation group did not show significant changes in the DMN's connectivity, highlighting a specific impact of active art production on brain function. Additionally, the visual art production group demonstrated increased specificity and differentiation in the sensorimotor cortex (S1/M1) at rest, suggesting improved brain function in regions involved in motor and sensory processing.

The findings suggest that engaging in visual art production can lead to enhancements in the functional connectivity of the brain, particularly within the mPFC and amygdala of the DMN, which is associated with cognitive processes like introspection and self-awareness. It is important to note that the study's findings are based on a small, non-clinical sample of post-retirement adults, limiting the generalizability of the results.

The observation that visual art production leads to increased functional connectivity within the DMN, especially between PCC, preCUN, and frontal and parietal cortices, supports the idea that engaging in creative activities can alter brain function in ways that may enhance ER. Since the DMN is associated with self-referential thought processes, an enhancement in its connectivity could facilitate more effective emotional processing and regulation.

Kreplin and Fairclough (2013) explored how the medial rostral prefrontal cortex (mrPFC; BA10) is activated during the viewing of visual arts that evoke positive and negative emotions. Thirty right-handed participants without formal art training were recruited to ensure that the study results were not biased by professional familiarity with visual art. The experimental setup involved viewing 16 pre-rated images (eight positive, eight negative) under two conditions: emotional introspection (EI) and spot-the-difference (SD), serving as a task for external object identification. The use of fNIRS allowed for the monitoring of brain activity, specifically changes in oxygenated and deoxygenated hemoglobin, as indicators of neural activation. The mean increase in oxygenated hemoglobin in the rPFC was 0.5 μM for positive images, compared to 0.2 μM for negative images (*p* < 0.05). The effect size for the difference between positive and negative images was Cohen's *d* = 0.65, indicating a moderate to large effect.

The findings revealed a significant increase in oxygenated blood in the medial rPFC when participants viewed positive images compared to negative ones, indicating a differential brain response based on the emotional valence of the artwork. Notably, there was no significant difference in brain activation between the two viewing conditions (EI and SD), suggesting that the emotional impact of the art was consistent regardless of the focus on internal feelings or external stimulus properties. One limitation noted is the potential confounding effect of having the art stimulus present during both conditions, possibly affecting the purity of internal vs. external attentional focus.

The study suggests that the mrPFC plays a significant role in the emotional evaluation of art, particularly in response to positive imagery. This neural activation pattern suggests a fundamental neural basis for the aesthetic appreciation of art that transcends the mere cognitive analysis of visual properties. The involvement of the medial rPFC in processing positive art evaluations and its role in ER provides neural evidence supporting the hypothesis that engagement in creative arts (like viewing art) may activate similar brain networks involved in adaptive ER.

Carlson et al. (2015) conducted a study on the behavioral and neural correlates of adaptive and maladaptive emotion regulation strategies through music in males and females. A total of 123 participants underwent psychological assessments to evaluate depression, anxiety, Neuroticism, and their uses of music for mood regulation (MMR). Of these, a subset of 56 participants also participated in an fMRI study to examine neural responses to music in the mPFC.

The study reported a significant gender difference in the neural response to music, with females showing a stronger correlation between mPFC activation and positive mood regulation (*r* = 0.55, *p* < 0.01). The effect size for the difference in mPFC activation between discharge and diversion strategies was Cohen's *d* = 0.78, indicating a large effect.

Behavioral results highlighted a positive correlation between the use of music as a discharge mechanism (expressing negative emotions through music) and increased levels of anxiety and Neuroticism, especially in males. This correlation suggests that employing music as a discharge strategy might be linked to maladaptive patterns of emotion regulation. Conversely, the diversion strategy (using music to distract from negative emotions) was associated with more adaptive regulation patterns, indicated by increased mPFC activity during music listening in females. This finding suggests gender-specific differences in how music listening strategies are employed and their neural underpinnings.

The study posits that while music has the potential to be a powerful tool for emotion regulation, the manner in which it is used can have significant implications for mental health. Specifically, the discharge strategy, particularly prevalent among males, may reinforce negative emotional states rather than alleviate them, potentially exacerbating issues like anxiety and Neuroticism. On the other hand, the diversion strategy, more common among females, appears to activate neural mechanisms that could contribute to more effective emotion regulation. Several limitations are acknowledged, such as the lack of measures for non-musical mood regulation behaviors and a focus solely on the mPFC, potentially providing an incomplete picture of the neural processes involved in music-induced emotion regulation. Additionally, the generalizability of the findings may be limited by the sample's cultural and age homogeneity.

The study's acknowledgments that different music listening strategies can activate the mPFC in gender-specific ways are especially relevant. These results contribute to our understanding of how the mPFC, a crucial area for emotion regulation, is involved in the emotional responses elicited by music listening. The gender differences observed in the neural responses to music listening strategies may inform further inquiries into how individual differences affect the engagement with creative arts for ER.

Limb and Braun (2008) conducted an fMRI study to explore the neural substrates of spontaneous musical performance, specifically examining jazz improvisation in professional pianists. The study identified a unique pattern of brain activity associated with the creative process of improvisation, characterized by a dissociated pattern of activity in the prefrontal cortex. This included deactivation of dmPFC (BA 8,9) medial dlPFC (BA 46), lateral dlPFC (BA9), superior dlPFC (BA8), ventral lateral orbitofrontal (lOFC) (BA 11, 47), and Mid lOFC (BA11) alongside focal activation of the mPFC (BA 10).

The study reported a 1.3% increase in BOLD signal in the mPFC during improvisation compared to the control task (*p* < 0.05). This activation was accompanied by a broader network activation including the sensorimotor cortices, with significant increases in BOLD signal observed in the precentral gyrus (BA 6, *z* = 3.12, *p* < 0.001).

Such a pattern suggests a combination of psychological processes necessary for spontaneous improvisation, highlighting a shift away from self-monitoring and conscious control toward a more free-flowing creative state. Furthermore, improvisation was associated with widespread activation of neocortical sensorimotor areas and deactivation of limbic structures, indicating a complex interplay between cognitive control, emotional regulation, and motor execution during creative musical expression. The study's findings highlight the significant neurobiological underpinnings of spontaneous creativity, emphasizing the role of specific prefrontal cortex regions in facilitating a state conducive to creative output. The research, however, was limited by its small sample size, focusing on six highly trained jazz musicians, which may not generalize across broader populations or different musical genres.

Finally, McPherson et al. (2016) investigated the interaction between the neural systems involved in creativity and those involved in emotion through an fMRI study of 12 professional jazz pianists. Participants were shown photographs of an actress depicting positive, ambiguous, and negative emotions. For each photograph, participants received visual and auditory cues instructing them to engage in one of three tasks: view the image, play a chromatic scale, or improvise music. The improvisation task required participants to create music on a custom-built, non-ferromagnetic piano keyboard that they felt represented the emotion in the photograph. This experimental design aimed to examine whether emotional intent influences the neural substrates of creativity.

BA 45 was activated in all emotion conditions (positive, negative, and ambiguous), while the Supplementary Motor Area (SMA) (BA 6) was activated for ambiguous and negative improvisation. Conversely, deactivations were observed in the Bilateral Angular Gyrus (AG), (BA 40), Middle Precuneus (BA 7), Medial and Lateral Frontopolar Prefrontal Cortex (BA 10), and dlPFC across all emotion conditions. Emotion-specific activations included the right superior temporal lobe and temporal pole (BA 22 and BA 21) during ambiguous improvisation, and more widespread deactivation in the dlPFC, AG, and precuneus during positive improvisation compared to negative and ambiguous conditions.

Between-emotion contrasts revealed heightened activity in the right insula (BA 13 and 47), right anterior cingulate cortex (BA 32), right parietal cortex (BA 40), bilateral middle temporal lobes (BA 22), and bilateral middle frontopolar prefrontal cortex (BA 10) during positive vs. negative and ambiguous improvisation. Positive improvisation also showed increased cerebellar activity and more activity in the left hippocampus, amygdala, and right parahippocampal gyrus.

The study observed a main effect of emotion on note densities [*F*_(1, 3)_ = 31.88, *p* < 0.001], with positive emotions being expressed through higher note densities (mean note density: positive = 3.61 notes/s, negative = 2.09 notes/s, ambiguous = 2.56 notes/s). Significant differences in connectivity were found using PPI analysis between the left insula and various regions, depending on the emotional context. For instance, the left insula showed significantly higher connectivity with the superior medial gyrus during positive improvisation compared to negative improvisation (*p* < 0.001).

The study found that emotional context significantly modulates brain activity in areas associated with creativity. Notably, the mPFC and limbic regions such as the amygdala and insula showed varying levels of activity and functional connectivity depending on the emotional intent behind the improvisation. Positive emotions elicited more extensive deactivation in the dlPFC, suggesting a deeper state of creative flow. Conversely, negative emotions were associated with heightened connectivity between the amygdala and areas involved in emotional processing and reward, indicating a different pathway of emotional influence on creativity. Musical improvisation, irrespective of emotional context, engaged brain regions known to be involved in language processing, motor planning, and sensory-motor feedback.

The study's findings underscore the tight integration between emotion and creativity, showing that the creative process in music is not just a cognitive task but also emotional. The findings suggest a dynamic neural model of creativity that is sensitive to emotional context. The differential activation and connectivity patterns observed highlight the role of the mPFC and limbic system in mediating the creative process under the influence of emotions. The study's limitations include its focus on a specific form of music creativity (musical improvisation) and its small sample size, which consisted of male participants.

The observed modulation of creativity-related brain networks by emotional intent, and the specific roles of the mPFC and limbic structures in this process, support the hypothesis of a shared neural mechanism between artistic creativity and ER.

## 4 Discussion

### 4.1 The role of the PFC and the amygdala in engagement and exposure to creative arts

This systematic review critically examined the evidence supporting the hypothesis of a common neuronal mechanism for ER and engagement in or exposure to artistic creations, with a focus on the roles played by the mPFC and the amygdala in both contexts. We found non-clinical fMRI and fNIRS studies assessing the neuronal correlates of creative engagement (Bolwerk et al., 2014; McPherson et al., 2016; Limb and Braun, 2008; Zhang et al., 2023), as well as non-clinical fMRI studies investigating the neuronal correlates of exposure to artistic exposure (Kreplin and Fairclough, 2013; Carlson et al., 2015).

While the studies included are diverse in terms of art type and engagement modality (creation vs. exposure), we find a consistent pattern of neural activation involving the prefrontal cortex. Some studies also report the engagement of the amygdala during artistic creation (Zhang et al., 2023) and artistic improvisation tasks (McPherson et al., 2016; Limb and Braun, 2008). These results suggest that the PFC appears to participate in both artistic production and exposure to at least certain art types (visual arts and music).

The findings above are consistent with several assumptions we made based on previous literature. For instance, previous literature has found the PFC (Raichle, 2015) and the amygdala (Chan et al., 2023) participate in creative activities, among other structures that belong to the DMN and CEN (Raichle, 2015; Bolwerk et al., 2014). We found that the mPFC and the amygdala participate in drawing (Zhang et al., 2023) and creative improvisation (McPherson et al., 2016; Limb and Braun, 2008), and the mPFC is also activated during exposure to music (Carlson et al., 2015). We also found that the PFC does not only participate in artistic creation but also in exposure to other creations, with the rPFC being activated during exposure to visual arts (Kreplin and Fairclough, 2013) and mPFC being activated during exposure to music (Carlson et al., 2015).

These findings advance the literature by suggesting that both active and passive engagement with artistic creations activate specific parts of the brain, including the PFC and amygdala. If we assume that activation of these brain regions is associated with improved ER, our results provide a compelling argument that engagement in or exposure to creative arts could offer clinical benefits.

### 4.2 Creative arts and ER

Our literature review shows that engagement and even exposure to creative arts activates parts of the brain that participate in ER. For instance, we know that the PFC plays an important role in adaptive and maladaptive emotional processing (Murakami et al., 2015; Gross and John, 2003) and we showed that this part of the brain is consistently activated during creative engagement/exposure.

We also know from previous research that adaptive ER strategies, such as reappraisal and mindfulness, engage the mPFC in conjunction with the amygdala (Chen et al., 2017). We also know that the amygdala also participates in the immediate emotional response to stimuli, particularly emotions related to fear or pleasure and its engagement is key for the emotional intensity required in creativity and for the embodied experience of emotions (Chen et al., 2017; Šimić et al., 2021; Bashwiner et al., 2016). We found that creative arts engage both structures simultaneously (McPherson et al., 2016; Zhang et al., 2023) during artistic creation, which suggests artistic creation may contribute to ER by activating structures that participate in it. On the other hand, we also found that improvisation activated the mPFC, but deactivated limbic structures, suggesting a complex interplay between cognitive control, emotional regulation, and motor execution during creative musical expression.

The identification of a common neuronal mechanism underlying engagement with or exposure to creative arts and ER highlights a potential avenue for enhancing clinical practices. Specifically, the findings suggest that creative arts could be a valuable tool in improving ER, thereby enhancing emotional intelligence and resilience. This insight supports the use of creative therapies as an effective approach to improving the quality of life for individuals with low ER capacity, offering a new dimension to mental health interventions.

Furthermore, the observed activation patterns in both the PFC and amygdala during creative engagement and exposure suggest potential biomarkers for evaluating the efficacy of art-based interventions in clinical settings.

### 4.3 Limitations of the study and future research directions

Our study comes with important limitations, which is to be expected given the lack of research investigating our hypothesis. Very few studies on fMRI studies have explored the relationship between engagement in artistic creation or exposure to artistic creations and ER. As such, our systematic review is limited by the small number of studies that were included. In addition, we also note methodological limitations encountered in the review, including variability in study design and population demographics, which may influence the generalizability of the findings.

Equally important, we need to distinguish between studies conducted in controlled laboratory settings and those in real-world clinical interventions such as randomized controlled trials (RCTs). Laboratory studies focus on specific aspects of the therapeutic process under controlled conditions, aiming to isolate and understand particular mechanisms or effects. In contrast, RCTs and real-world interventions assess the overall efficacy of creative arts therapies within complex, real-life clinical settings, considering various interacting factors. We acknowledge there may be differences in neuronal response between laboratory studies and clinical interventions. We also acknowledge that the effects of engagement might be different depending on whether individuals engage with creative arts by themselves or in a group.

Finally, it's important to note that while creativity is a potential major mechanism underlying the therapeutic effects of engagement with creative arts, other mechanisms may contribute to these benefits and separating the effects of each mechanism is a challenging task, to say the least. In visual art-making, the tactile interaction with art materials (e.g., clay, paint, and textiles) will engage sensory and motor pathways and this sensorial experience may enhance emotional expression and regulation (Wong and Au, 2019). In dance and movement therapy, the physical movement of the body is a central component. This movement can help release pent-up emotions, increase body awareness, and improve mood through the release of endorphins. The aesthetic experience itself, whether through creating or observing art, can evoke powerful emotional responses. This experience can activate the brain's reward system, providing a sense of pleasure and satisfaction (Ritter and Low, 1996). The appreciation of beauty in art can also stimulate cognitive and emotional processes, contributing to enhanced wellbeing.

To address some of these limitations and build upon the current findings, future research should aim to standardize methodologies across studies to facilitate comparability and replication. Hybrid studies that compare outcomes between different conditions can clarify the nature of the potential differences in neuronal responses between laboratory studies and clinical interventions. Longitudinal studies are needed to assess the durability of the neural and psychological changes induced by creative arts interventions.

Studies should also explore how different modalities of creative arts therapies can be tailored to individual needs and conditions, and how these interventions can be integrated with existing therapeutic practices. Finally, future studies should seek to explore the potential differences in clinical effects and neuronal correlates between engagement with and exposure to the creative arts.

## 5 Conclusion

This systematic review examined the neuroanatomical basis of ER and creative engagement to determine whether they share common underlying neuronal mechanisms. The recognition of a common mechanism highlights the potential of creative arts as a complementary therapy for conditions characterized by low ER. We found consistent activation of the mPFC and amygdala during creative engagement, suggesting that these regions are involved in both adaptive ER and creative processes. These findings support the hypothesis that creative arts may engage similar neural networks as those used in ER, offering a neuroscientific basis for the observed benefits of creative therapies in enhancing emotional intelligence and facilitating emotional processing. However, the study's limitations, including a small number of studies, a lack of clinical trials, and methodological constraints, suggest that further research is needed. Future studies should focus on standardizing methodologies, conducting longitudinal research, and exploring the specific mechanisms through which creative arts contribute to ER, which could inform the development of more effective therapeutic interventions.

## Data Availability

The original contributions presented in the study are included in the article/supplementary material, further inquiries can be directed to the corresponding authors.
